# Supplementary material for: Effect of Sustainably Sourced Protein Consumption on Nutrient Intake and Gut Health in Older Adults: A Systematic Review
Source: Nutrients. 2024 May 6;16(9):1398. doi: 10.3390/nu16091398 (PMC11085519; doi:10.3390/nu16091398)
Supplement: Supplementary file 1 [file nutrients-16-01398-s001.zip › nutrients-2981279-supplementary.pdf]

Supplementary Table S1: Prisma Checklist 2020

Section and Topic	Item	Checklist item	Location where item is reported
**TITLE**			
Title	1	Identify the report as a systematic review.	Title, Pg1
**ABSTRACT**			
Abstract	2	See the PRISMA 2020 for Abstracts checklist.	Pg2, lines 13 - 28
**INTRODUCTION**			
Rationale	3	Describe the rationale for the review in the context of existing knowledge.	Pg1-3
Objectives	4	Provide an explicit statement of the objective(s) or question(s) the review addresses.	Pg3, lines 127-133
**METHODS**			
Eligibility criteria	5	Specify the inclusion and exclusion criteria for the review and how studies were grouped for the syntheses.	Pg3-4, lines 135-190 and Supp Table S2
Information sources	6	Specify all databases, registers, websites, organisations, reference lists and other sources searched or consulted to identify studies. Specify the date when each source was last searched or consulted.	Pg4-5, lines 191-203 and Supp Table S3
Search strategy	7	Present the full search strategies for all databases, registers and websites, including any filters and limits used.	Pg5, lines 204-208 and Supp Figure S1
Selection process	8	Specify the methods used to decide whether a study met the inclusion criteria of the review, including how many reviewers screened each record and each report retrieved, whether they worked independently, and if applicable, details of automation tools used in the process.	Pg5, lines 209-216
Data collection process	9	Specify the methods used to collect data from reports, including how many reviewers collected data from each report, whether they worked independently, any processes for obtaining or confirming data from study investigators, and if applicable, details of automation tools used in the process.	Pg5, lines 217-224
Data items	10a	List and define all outcomes for which data were sought. Specify whether all results that were compatible with each outcome domain in each study were sought (e.g. for all measures, time points, analyses), and if not, the methods used to decide which results to collect.	Pg5, lines 225-246
	10b	List and define all other variables for which data were sought (e.g. participant and intervention characteristics, funding sources). Describe any assumptions made about any missing or unclear information.	Pg5-6, lines 247-251
Study risk of bias assessment	11	Specify the methods used to assess risk of bias in the included studies, including details of the tool(s) used, how many reviewers assessed each study and whether they worked independently, and if applicable, details of automation tools used in the process.	Pg6, lines 252-264
Effect measures	12	Specify for each outcome the effect measure(s) (e.g. risk ratio, mean difference) used in the synthesis or presentation of results.	Pg6, lines 265-269
Synthesis methods	13a	Describe the processes used to decide which studies were eligible for each synthesis (e.g. tabulating the study intervention characteristics and comparing against the planned groups for each synthesis (item #5)).	Narrative review
	13b	Describe any methods required to prepare the data for presentation or synthesis, such as handling of missing summary statistics, or data conversions.	Narrative review
	13c	Describe any methods used to tabulate or visually display results of individual studies and syntheses.	Narrative review
	13d	Describe any methods used to synthesize results and provide a rationale for	Narrative

Section and Topic	Item	Checklist item	Location where item is reported
		the choice(s). If meta-analysis was performed, describe the model(s), method(s) to identify the presence and extent of statistical heterogeneity, and software package(s) used.	review
	13e	Describe any methods used to explore possible causes of heterogeneity among study results (e.g. subgroup analysis, meta-regression).	Narrative review
	13f	Describe any sensitivity analyses conducted to assess robustness of the synthesized results.	Narrative review
Reporting bias assessment	14	Describe any methods used to assess risk of bias due to missing results in a synthesis (arising from reporting biases).	Pg6, lines 267-268
Certainty assessment	15	Describe any methods used to assess certainty (or confidence) in the body of evidence for an outcome.	Pg6, lines 267-268
**RESULTS**			
Study selection	16a	Describe the results of the search and selection process, from the number of records identified in the search to the number of studies included in the review, ideally using a flow diagram.	Pg6, lines 271-281 and Fig 1
	16b	Cite studies that might appear to meet the inclusion criteria, but which were excluded, and explain why they were excluded.	Pg6, lines 278-279 and Fig 1
Study characteristics	17	Cite each included study and present its characteristics.	Pg7-10, lines 284-340 and Tables 1&2
Risk of bias in studies	18	Present assessments of risk of bias for each included study.	Pg12-13, lines 350-390 and Table 3
Results of individual studies	19	For all outcomes, present, for each study: (a) summary statistics for each group (where appropriate) and (b) an effect estimate and its precision (e.g. confidence/credible interval), ideally using structured tables or plots.	Pg13-20, lines 391-563,Tables 4,5&6 and Supp Table S4
Results of syntheses	20a	For each synthesis, briefly summarise the characteristics and risk of bias among contributing studies.	Narrative review
	20b	Present results of all statistical syntheses conducted. If meta-analysis was done, present for each the summary estimate and its precision (e.g. confidence/credible interval) and measures of statistical heterogeneity. If comparing groups, describe the direction of the effect.	Narrative review
	20c	Present results of all investigations of possible causes of heterogeneity among study results.	Narrative review
	20d	Present results of all sensitivity analyses conducted to assess the robustness of the synthesized results.	Narrative review
Reporting biases	21	Present assessments of risk of bias due to missing results (arising from reporting biases) for each synthesis assessed.	Narrative review
Certainty of evidence	22	Present assessments of certainty (or confidence) in the body of evidence for each outcome assessed.	Narrative review
**DISCUSSION**			
Discussion	23a	Provide a general interpretation of the results in the context of other evidence.	Pg20-22, lines 564-676
	23b	Discuss any limitations of the evidence included in the review.	Pg22, lines 677-689
	23c	Discuss any limitations of the review processes used.	Pg22, lines 677-689
	23d	Discuss implications of the results for practice, policy, and future research.	Pg22, lines 690-702
**OTHER INFORMATION**			

Section and Topic	Item	Checklist item	Location where item is reported
Registration and protocol	24a	Provide registration information for the review, including register name and registration number, or state that the review was not registered.	Pg22, lines 703-707
	24b	Indicate where the review protocol can be accessed, or state that a protocol was not prepared.	Pg22, lines 703-707
	24c	Describe and explain any amendments to information provided at registration or in the protocol.	Pg22, lines 703-707
Support	25	Describe sources of financial or non-financial support for the review, and the role of the funders or sponsors in the review.	Pg22, lines 717-718
Competing interests	26	Declare any competing interests of review authors.	Pg23, line 725
Availability of data, code and other materials	27	Report which of the following are publicly available and where they can be found: template data collection forms; data extracted from included studies; data used for all analyses; analytic code; any other materials used in the review.	Pg23, lines 719-722, Tables 1-6 and supplementary material

From: Page MJ, McKenzie JE, Bossuyt PM, Boutron I, Hoffmann TC, Mulrow CD, et al. The PRISMA 2020 statement: an updated guideline for reporting systematic reviews. BMJ 2021;372:n71. doi: 10.1136/bmj.n71

Supplementary Table S2: Inclusion and exclusion criteria for screening articles

The aim of this systematic review is to evaluate the literature on the effect of consumption of sustainable proteins compared to non-sustainable or meat-based proteins on nutrient intake, gut health and age-related changes in healthy, community-dwelling individuals aged 65 years or over.

	**Inclusion criteria**	**Exclusion criteria**
**Population**		
Age	65 years and older (also include studies addressing adults in general, if data for older adults is reported separately).	Children, adolescents, young and middle aged adults.
Disease	Healthy, Under/overweight, or obese	Studies restricted to specific diseases, conditions, or metabolic disorders
**Intervention**		
Dietary	• Consumption of sustainable proteins and diets, plant-based proteins, vegan, vegetarian, Mediterranean, legumes or pulses, soy, wheat, vegetables, potatoes, beans, peas, quinoa, amaranth, buckwheat, rapeseed oil, edible insects, algae (seaweed), microalgae (spirulina), aquatic plants (duckweed).<br>• Decreased consumption of red meat with increased consumption of sustainable proteins<br>• Educational or public health policy that increases consumption of sustainable diets	No dietary intervention or a dietary intervention that isn't considered sustainable/plant based.
Non-dietary	Intervention of other lifestyle component, e.g., physical activity but must include sustainable diet component.	Intervention that isn't lifestyle related
	2 weeks or more	Less than 2 weeks

Intervention period		
**Comparators**	• Low intake of sustainable or plant-based proteins<br>• High intake of animal-based proteins<br>• Certain type of diet (western)<br>• No educational or policy that increase consumption of sustainable diets	
**Outcomes**	• Nutrient intake<br>• Food group intake<br>• Gut microbiome status<br>• Health status	
**Settings**	• Community dwelling	• Hospital<br>• Care home/residential care
**Date and language**	No restrictions	
**Study design**	• RCTs (including cluster RCTs)<br>• Controlled (non-randomized) clinical trials<br>• Cluster trials<br>• Interrupted time series (ITS) studies with at least three data points before and after the intervention<br>• Controlled before-after (CBA) studies prospective and retrospective comparative cohort studies<br>• Observational studies<br>• Cross-sectional studies<br>• Case-control or nested case-control studies Cluster randomized, cluster non-randomized, or CBA studies will be included only if there are at least two intervention sites and two control sites	• Case series<br>• Case reports<br>• Studies in animals

Supplementary Table S3: Database names and dates of coverage

**Database**	**Coverage**
Ovid<br>Medline<br>APA PsycInfo<br>EBM Reviews – Cochrane CRCT<br>Embase	 1946 – present<br>1806 – present<br>July 23<br>1974 – present
EBSCOhost<br>CINAHL Plus	 1937 – present
Clarivate<br>Web of Science	 1900 – present

Supplementary Figure S1: Search strategies for all databases and trial registries

Medline, PsycInfo, EBM Reviews (Cochrane Central Registry of Controlled trials), and Embase (Ovid platform)

1. (Proteins or Dietary Proteins or Plant Proteins or Plant Proteins Dietary or Soy Proteins or Soybean Proteins or Animal protein or Fruit Proteins or Grain Proteins or Nut Proteins or Pea Proteins or Plants Edible).mp. [mp=title, book title, abstract, original title, name of substance word, subject heading word, floating sub-heading word, keyword heading word, organism supplementary concept word, protocol supplementary concept word, rare disease supplementary concept word, unique identifier, synonyms, population supplementary concept word, anatomy supplementary concept word]
2. (diet, vegetarian or Diet, vegan or Diet, Mediterranean or Diet, plant-based).mp.
3. (plant-based protein or meat-based protein or dairy-based protein or seafood-based protein or insect-based protein or single-cell protein or in vitro meat-based protein or cultured meat or in vitro meat or synthetic meat).mp.
4. (sustainable or sustainable diet* or alternative diet*).mp.
5. (older* or ageing or ageing or over 50 or elderly or aged).mp.
6. (gut health or microbiome or microbiota or gastrointestinal microbiome).mp.
7. 1 or 2 or 3 or 4
8. 5 and 6 and 7
9. limit 8 to humans

CINAHL Plus (EBSCOhost platform)

1. TX Proteins or Dietary Proteins or Plant Proteins or Plant Proteins Dietary or Soy Proteins or Soybean Proteins or Animal protein or Fruit Proteins or Grain Proteins or Nut Proteins or Pea Proteins or Plants Edible
2. TX diet, vegetarian or Diet, vegan or Diet, Mediterranean or Diet, plant-based
3. TX plant-based protein or meat-based protein or dairy-based protein or seafood-based protein or insect-based protein or single-cell protein or in vitro meat-based protein or cultured meat or in vitro meat or synthetic meat
4. TX sustainable or sustainable diet* or alternative diet*
5. TX older* or ageing or ageing or over 50 or elderly or aged
6. TX gut health or microbiome or microbiota or gastrointestinal microbiome
7. S1 OR S2 OR S3 OR S4
8. S5 AND S6 AND S7

Web of Science (Clarivate platform)

1. ALL=(Proteins or Dietary Proteins or Plant Proteins or Plant Proteins Dietary or Soy Proteins or Soybean Proteins or Animal protein or Fruit Proteins or Grain Proteins or Nut Proteins or Pea Proteins or Plants Edible)
2. ALL=(diet, vegetarian or Diet, vegan or Diet, Mediterranean or Diet, plant-based)
3. ALL=(plant-based protein or meat-based protein or dairy-based protein or seafood-based protein or insect-based protein or single-cell protein or in vitro meat-based protein or cultured meat or in vitro meat or synthetic meat)
4. ALL=(sustainable or sustainable diet* or alternative diet*)
5. ALL=(older* or ageing or ageing or over 50 or elderly or aged)
6. ALL=(gut health or microbiome or microbiota or gastrointestinal microbiome)
7. #1 OR #2 OR #3 OR #4
8. #5 AND #6 AND #7
9. #5 AND #6 AND #7 and Veterinary Sciences or Zoology (Exclude – Research Areas) and 3.51 Dairy & Animal Sciences or 3.232 Veterinary Sciences or 3.35 Zoology & Animal Ecology or 3.274 Animal Sensing (Exclude – Citation Topics Meso)

ISRCTN registry

- Search terms: Plant proteins OR sustainable
- Age range: Adult

ClinicalTrials.gov

- Condition or disease: Gut health OR Microbiota OR Microbiome
- Intervention/Treatment: Sustainable diet OR Plant protein
- Sex: All
- Age: Older adult (65+)

Supplementary Table S4: Health status outcomes

**Author, Year**	**BMI (kg/m^2)**	**Self-rated health, n (%)**	**Non-communicable diseases, n (%)**	**Cholesterol**	**Muscle mass (kg)**	**Grip strength (kg)**
André, 2021	By tertile of 3-OH FAs<br>n (%):<br>Under wt, <18.5:<br>Total: 6 (0.9)<br>T1: 0 (0.0)<br>T2: 1 (0.4)<br>T3: 5 (2.2)<br><br>Normal wt, 18.5–25:<br>Total: 264 (37.8)<br>T1: 98 (42.2)<br>T2:84 (36.1)<br>T3:82 (35.2)<br><br>Over wt, >25:<br>Total: 428 (61.3)<br>T1: 134 (57.8)	By tertile of 3-OH FAs<br><br>Good/very good:<br>Total: 21 (3)<br>T1: 7 (3.0)<br>T2: 7 (3.0)<br>T3: 7 (3.0)<br><br>Intermediate:<br>Total: 239 (34.2)<br>T1: 85 (36.6)<br>T2: 78 (33.5)<br>T3: 76 (32.6)<br><br>Poor/very poor:<br>Total: 438 (62.8)<br>T1: 140 (60.3)	By tertile of 3-OH FAs<br><br>Diabetes:<br>Total: 50 (7.2)<br>T1: 8 (3.5)<br>T2: 11 (4.7)<br>T3: 31 (13.3)<br><br>Met syndrome:<br>Total: 102 (14.9)<br>T1: 26 (11.4).<br>T2: 29 (12.7)<br>T3: 47 (20.7)<br><br>Hypertension:<br>Total: 529 (75.8)<br>T1: 168 (72.4)	By tertile of 3-OH FAs<br><br>Mean (SD), (Mmol/L):<br>LDL:<br>Total: 3.6 (0.9)<br>T1: 3.6 (0.8)<br>T2: 3.6 (0.8)<br>T3: 3.7 (0.9)<br><br>HDL:<br>Total: 1.6 (0.4)<br>T1: 1.6 (0.4)<br>T2: 1.6 (0.4)<br>T3: 1.6 (0.4)	Not reported	Not reported

	T2: 148 (63.5)<br>T3: 146 (62.6)	T2: 148 (63.5)<br>T3: 150 (64.4)	T2: 174 (74.7)<br>T3: 187 (80.3)<br><br>Cardio diseases:<br>Total: 55 (7.9)<br>T1: 12 (5.2)<br>T2: 18 (7.7)<br>T3: 25 (10.7)<br><br>Depression:<br>Total: 42 (6.0)<br>T1: 16 (6.9)<br>T2: 14 (6.0)<br>T3: 12 (5.2)			
Farsijani, 2022	By quartile, mean (SD):<br>Q1: 27.0 (3.8)<br>Q2: 27.1 (3.7)<br>Q3: 27.1 (3.6)<br>Q4: 26.8 (4.0)	By quartile, Excellent:<br>Q1: 75 (38.7)<br>Q2: 69 (35.8)<br>Q3: 68 (35.1)<br>Q4: 68 (35.1)	By quartile, Diabetes:<br>Q1: 22 (11.3)<br>Q2: 28 (14.5)<br>Q3: 34 (17.5)<br>Q4: 31 (16.0)<br><br>Hypertension:<br>Q1: 98 (50.5)<br>Q2: 98 (50.8)<br>Q3: 106 (54.6)<br>Q4: 101 (52)<br><br>Depression score (0-15):<br>Q1: 1.8 (2.0)<br>Q2: 1.7 (1.8)<br>Q3: 1.8 (1.8)<br>Q4: 1.4 (1.8)	Not reported	By quartile, Mean (SD)<br>Q1: 49.1 (5.6)<br>Q2: 58.6 (1.6)<br>Q3: 64.3 (1.9)<br>Q4: 76.0 (7.0)	By quartile, Mean (SD)<br>Q1: 36.4 (7.9)<br>Q2: 36.0 (7.4)<br>Q3: 35.8 (7.6)<br>Q4: 35.9 (7.6)
Ghosh, 2020	Median (min-max) at baseline<br><br>Control:<br>26.8 (18.8 - 44.6)<br>Diet group:<br>26.9 (18.5 - 46.0)	Not reported	Not reported	Not reported	Not reported	Not reported
Li, 2021	By quintile, mean (SD):<br>Q1: 26.2 (4.0)<br>Q2: 25.6 (3.4)<br>Q3: 24.5 (3.6)<br>Q4: 24.8 (2.5)<br>Q5: 23.9 (3.1)	Not reported	Not reported	Not reported	Not reported	Not reported
Maroto-Rodriguez, 2022	By tertile, n (%)<br>T1:<br><25: 71 (16.55)<br>25-29.9: 212 (49.42)<br>≥30: 146 (34.03)<br><br>T2:<br><25: 150 (19.61)<br>25-29.9: 391 (51.11)<br>≥30: 334 (48.69)<br><br>T3:<br><25: 147 (21.43)<br>25-29.9: 334 (48.69)<br>≥30: 205 (29.88)<br><br>Total sample, n (%):<br><25: 368 (19.57)<br>25-29.9: 937 (49.69)<br>≥30: 575 (30.59)	Not reported	By tertile, Cardio disease:<br>T1: 22 (5.13)<br>T2: 39 (5.10)<br>T3: 36 (5.25)<br><br>Type 2 diabetes:<br>T1: 74 (17.25)<br>T2: 117 (15.29)<br>T3: 93 (13.56)<br><br>Cancer:<br>T1: 9 (2.10)<br>T2: 13 (1.70)<br>T3: 12 (1.75)<br><br>Chronic lung disease:<br>T1: 24 (5.59)<br>T2: 68 (8.89)	Not reported	Not reported	Not reported

			T3: 51 (7.43)<br><br>Depression:<br>T1: 33 (7.69)<br>T2: 51 (6.67)<br>T3: 54 (7.87)			
Maskarinec, 2019	By tertile, mean (SD)<br>HEI-2020<br>T1: 21.9 (4.9)<br>T3: 26.6 (4.6)<br><br>AHEI-2010<br>T1: 29.1 (5.0)<br>T3: 26.7 (4.5)<br><br>aMED<br>T1: 28.6 (5.0)<br>T3: 27.3 (4.7)<br><br>DASH<br>T1: 29.0 (5.0)<br>T3: 23.5 (8.6)	Not reported	History of diabetes:<br>HEI-2020<br>T1: 214 (37)<br>T3: 168 (29)<br><br>AHEI-2010<br>T1: 197 (34)<br>T3: 156 (27)<br><br>aMED<br>T1: 276 (43)<br>T3: 116 (25)<br><br>DASH<br>T1: 240 (38)<br>T3: 140 (25)	Not reported	Not reported	Not reported
Shikany, 2019	By quartile, mean (SD)<br><br>Western diet<br>Total: 26.9 (3.7)<br>Q1: 26.0 (3.4)<br>Q2: 26.5 (3.6)<br>Q3: 27.4 (4.0)<br>Q4: 27.9 (3.6)<br><br>Prudent diet<br>Total: 26.9 (3.7)<br>Q1: 27.3 (3.5)<br>Q2: 27.3 (3.6)<br>Q3: 27.1 (4.0)<br>Q4: 26.0 (3.7)	By quartile, excellent health n(%)<br><br>Western diet<br>Total: 464 (89.7)<br>Q1: 120 (92.3)<br>Q2: 115 (89.2)<br>Q3: 120 (93.0)<br>Q4: 109 (84.5)<br><br>Prudent diet<br>Total: 467 (89.3)<br>Q1: 114 (87.7)<br>Q2: 121 (93.8)<br>Q3: 116 (89.9)<br>Q4: 113 (87.6)	History of multimorbidity, n(%)<br><br>Western diet<br>Total: 322 (62.3)<br>Q1: 77 (59.2)<br>Q2: 80 (62.0)<br>Q3: 80 (62.0<br>Q4: 85 (65.9)<br><br>Prudent diet<br>Total: 322 (0.61)<br>Q1: 81 (62.3)<br>Q2: 92 (71.3)<br>Q3: 69 (53.5)<br>Q4: 80 (62.0)	Not reported	Not reported	Not reported
Trichopoulou, 2003	Not reported	Not reported	>65 years sub-group, No. of deaths/No. of person years:<br>Med T1: 80/5836<br>Med T2: 62/6889<br>Med T3: 32/3558<br><br>>55 years sub-group, No. of deaths/No. of participants:<br>229/9398	Not reported	Not reported	Not reported
Zhang, 2021	By timepoint, mean (SD)<br>Baseline: 24.0 (2.8)<br>Month 2: 24.3 (3.1)<br>Month 4: 24.3 (3.2)	Not reported	Not reported	At baseline, month 2 and month 4, mean (SD) (mg/dL)<br><br>Total cholesterol:<br>185.1 (42.0)<br>191.9 (46.4)<br>191.8 (37.5)<br><br>LDL:	Not reported	Not reported

				102.0 (34.7)<br>110.3 (38.3)<br>103.2 (30.0)<br><br>HDL:<br>61.2 (12.1)<br>68.4 (12.4)<br>61.8 (14.8)		

wt: weight, Met: Metabolic, Q: quintile/quartile, Cardio: Cardiovascular, T: tertile, HEI-2010: Healthy Eating Index 2010, AHEI-2010: Alternative Healthy Eating Index 2010, aMED: Alternate Mediterranean Diet, DASH diet: Dietary Approaches to Stop Hypertension Trial diet, No.: number,
